# Health service utilisation by quota, family-sponsored and convention refugees in their first five years in New Zealand

**DOI:** 10.23889/ijpds.v9i5.2762

**Published:** 2024-09-18

**Authors:** Frederieke Petrović-van der Deen, Jonathan Kennedy, James Stanley, Arezoo Malihi, Sheree Gibb, Ruth Cunningham

**Affiliations:** 1 University of Otago; 2 University of Auckland

## Objective and Approach

This study aims to explore and compare health service use between New Zealand’s (NZ) three main refugee groups and the general population. The StatsNZ’s Integrated Data Infrastructure (IDI) is a large collection of deidentified whole population administrative and survey datasets, linked at the individual level. Within the IDI, visa and border movement data was used to identify quota, family-sponsored and convention refugees who arrived in NZ in 2007-2013. Linked health data records were then used to explore primary care enrolment and consultations, emergency department (ED) presentations and specialist mental health service use in the first five years in NZ. Health service use patterns were compared between refugee groups and to the general population using logistic regression models, adjusted for age, sex, and deprivation.

## Results

Quota refugees were more likely to be enrolled and in contact with primary care and mental health services in their first year in NZ than family-sponsored and convention refugees, but differences reduced in subsequent post-arrival years. All refugee groups were more likely than the general NZ population to have presented to ED in their first year in NZ. Study findings have been published: https://pubmed.ncbi.nlm.nih.gov/37301053/.

## Results

Quota refugees were better connected with health services in their first year in NZ than the other two refugee groups. Refugee groups accessed different types of frontline health services than the general population.

## Implications

NZ would benefit from a consistent nationwide approach wherein all refugees receive equal health and settlement support regardless of their visa type or settlement location.

